# Interkingdom Gut Microbiome and Resistome of the Cockroach *Blattella germanica*

**DOI:** 10.1128/mSystems.01213-20

**Published:** 2021-05-11

**Authors:** Rebeca Domínguez-Santos, Ana Elena Pérez-Cobas, Paolo Cuti, Vicente Pérez-Brocal, Carlos García-Ferris, Andrés Moya, Amparo Latorre, Rosario Gil

**Affiliations:** a Institute for Integrative Systems Biology (I^2^SysBio), University of Valencia and CSIC, Valencia, Spain; b Institut Pasteur, Biologie des Bactéries Intracellulaires, Paris, France; c Genomics and Health Area, Foundation for the Promotion of Sanitary and Biomedical Research (FISABIO), Valencia, Spain; d Biomedical Research Center Network of Epidemiology and Public Health (CIBEResp), Madrid, Spain; e Department of Biochemistry and Molecular Biology, University of Valencia, Valencia, Spain; University of Hawaii at Manoa

**Keywords:** *Blattella germanica*, symbiosis, gut microbiome, kanamycin, antibiotic resistance genes (ARGs)

## Abstract

Cockroaches are intriguing animals with two coexisting symbiotic systems, an endosymbiont in the fat body, involved in nitrogen metabolism, and a gut microbiome whose diversity, complexity, role, and developmental dynamics have not been fully elucidated. In this work, we present a metagenomic approach to study Blattella germanica populations not treated, treated with kanamycin, and recovered after treatment, both naturally and by adding feces to the diet, with the aim of better understanding the structure and function of its gut microbiome along the development as well as the characterization of its resistome.

**IMPORTANCE** For the first time, we analyze the interkingdom hindgut microbiome of this species, including bacteria, fungi, archaea, and viruses. Network analysis reveals putative cooperation between core bacteria that could be key for ecosystem equilibrium. We also show how antibiotic treatments alter microbiota diversity and function, while both features are restored after one untreated generation. Combining data from B. germanica treated with three antibiotics, we have characterized this species’ resistome. It includes genes involved in resistance to several broad-spectrum antibiotics frequently used in the clinic. The presence of genetic elements involved in DNA mobilization indicates that they can be transferred among microbiota partners. Therefore, cockroaches can be considered reservoirs of antibiotic resistance genes (ARGs) and potential transmission vectors.

## Abstract (precis)

For the first time, we analyze the interkingdom hindgut microbiome of this species, including bacteria, fungi, archaea, and viruses. Network analysis reveals putative cooperation between core bacteria that could be key for ecosystem equilibrium.

## INTRODUCTION

Symbiosis is a widespread phenomenon in nature, and its relevance in evolution has been widely demonstrated, insects being one of the most studied groups. Many insects harbor a complex microbiome playing important roles for the host (1–3), being the gut microbiota one of the more intensely studied. Additionally, about 15% of insects have established an endosymbiotic relationship with one (or few) bacteria. Endosymbionts live in specialized host cells. Its main role is to provide the nutrients that are lacking in the unbalanced insect diet and that can also participate in nitrogen recycling (reviewed in references [Bibr B4][Bibr B5][Bibr B6]). The gut microbiota are microbial communities with a known contribution to host biology (7–10).

Many gut microbiomes have been characterized in different insect orders, such as termites (11, 12) and cockroaches (13–17) in Blattodea, crickets (18) and beetles (19–21) in Coleoptera, bees (22–25) in Hymenoptera, fruit fly (1, 26, 27) in Diptera, and several Lepidoptera (28, 29). Most studies focus on bacterial communities, even though other microbes (i.e., fungi, archaea, and viruses) cohabit within them (30–34). It is long known that Ascomycota and Basidiomycota fungi are an essential part of the community life of social insects such as ants and mound-building termites, which maintain fungal gardens as their primary food source (35–37). Both methanogenic and nonmethanogenic archaea have been reported in beetles, cockroaches, termites, and millipedes (30, 38). Finally, viruses belonging to several families have commonly been associated with insects, and new insect viruses have been discovered through metagenomic approaches (30, 39). Nevertheless, studies focusing on all microbiome members of the insect’s gut are lacking.

Cockroaches are a paradigmatic symbiotic case. Each insect harbors two symbiotic systems, the endosymbiont *Blattabacterium* in the fat body, and a complex microbial community in the hindgut. *Blattabacterium* genomic studies in many cockroach species demonstrated its essential role in nitrogen metabolism (40–46), whereas the gut microbiome’s role remains unclear. The composition of the gut bacterial community of the German cockroach Blattella germanica was first determined by 16S rRNA gene analysis in lab-reared and field-collected cockroaches (14, 47). Additional studies were made in populations reared with diets differing in protein content (48) and treated with rifampicin (49). Recently, the metagenomic characterization of two antibiotic-treated (vancomycin and ampicillin) populations and their comparison to control conditions shed light on the gut bacterial community’s function and ecology, as well as the mechanism for its acquisition after hatching (13). The above-mentioned studies use different approaches (i.e., 16S rDNA sequencing and whole-genome sequencing) to analyze the microbiota composition, and slightly different results can be obtained mainly for rare groups or taxa depending on 16S primers' specificity (50). Nevertheless, their results are comparable regarding the most abundant taxa, which are correctly detected by both methodologies (13). Thus, altogether, these previous studies revealed a stable adult core microbiome, capable of high resilience after a generation of antibiotic treatment. However, the putative existence of a nymphal core microbiome was not analyzed.

On the other hand, there is a growing concern about the antibiotic resistance of pathogenic microbes. The use of antibiotics in human and animal health, agriculture, aquaculture, or wastewater treatments, together with the poor knowledge of how to remove them from the environment, has led to a persistent antibiotic presence (51, 52). Metagenomic studies allow discovering the antibiotic resistance genes (ARGs) carried by microbiomes, something particularly interesting in insects with clinical, environmental, and economic importance (13, 53, 54). This is the case for cockroaches, with large population sizes in and around houses, hospitals, and in unsanitary and insalubrious areas. B. germanica is one of the few animal species living in close contact with humans and capable of exploiting always-changing urban environments. Thus, it is an appropriate model to study ARGs, since members of its gut microbial community could carry them, making this species a possible vector or reservoir (13).

In the present work, we have performed a metagenomic study of the gut microbiome of *B. germanica* in two generations of parallel-reared untreated and kanamycin-treated populations, in two nymphal stages and three adult time points. Our main goals were (i) to analyze the diversity and functions of its microbiome along the development, including bacteria, fungi, archaea, and viruses; (ii) to test the resilience of the microbiome through two generations of antibiotic treatment and after its cessation, and (iii) to characterize its resistome.

## RESULTS

### Composition and diversity of the gut microbiome of *B. germanica*.

**FIG 1:**
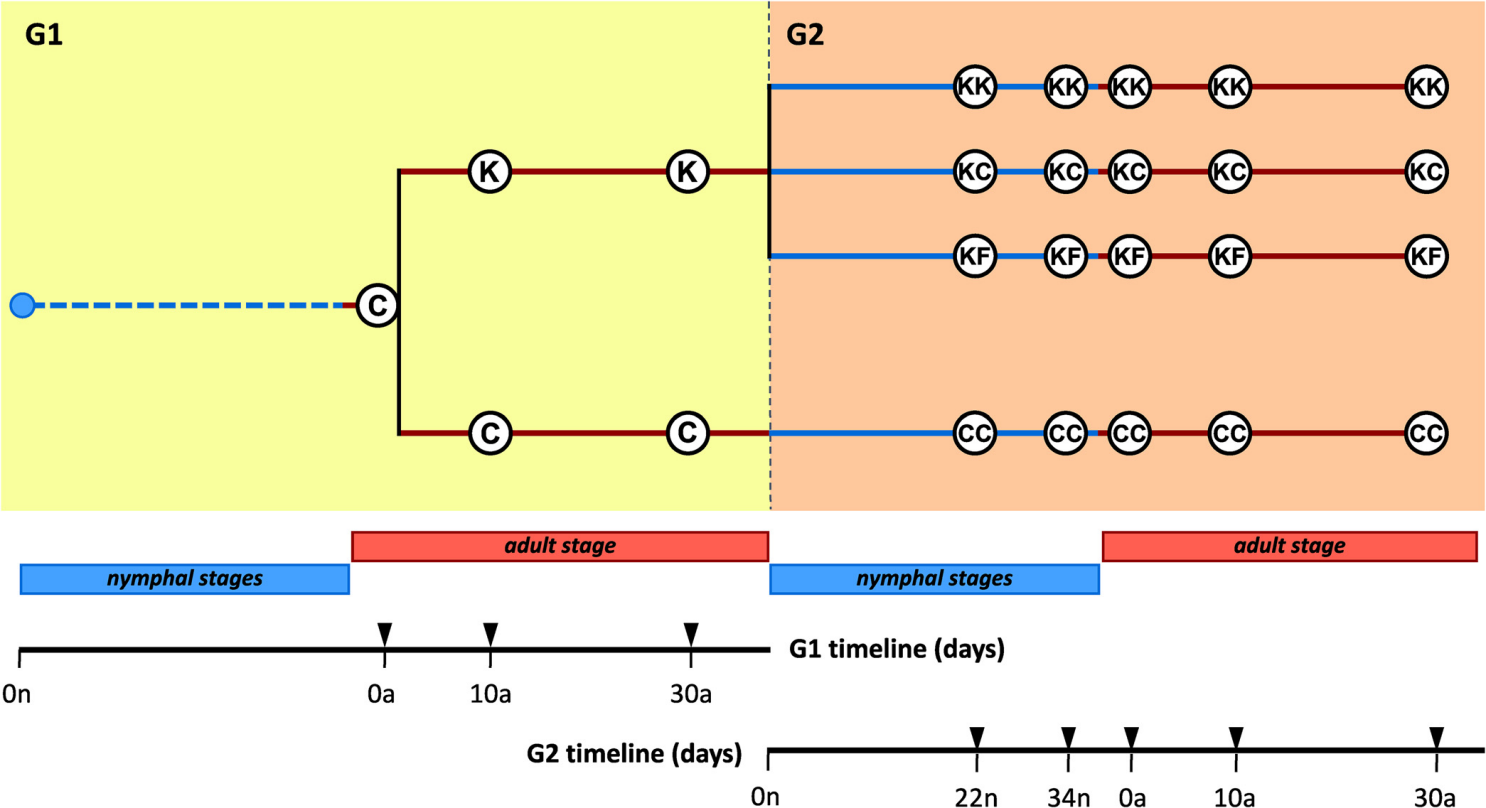
Experimental design. The antibiotic kanamycin was applied to a synchronized adult population of *B. germanica* in two generations (G1 and G2). At G1, two populations were established: a control one without antibiotic (C) and another one treated with kanamycin (K). At G2, the nymphs from the antibiotic-treated population were used to start three new populations: treated with kanamycin (KK), antibiotic-free (KC), or antibiotic-free with feces added to the diet (KF). The nymphs from the control population at G1 (C) were used to establish the G2 control population (CC). The numbers on the timelines indicate the time (in days) of the nymphal stage (n, blue line) or adult stage (a, red line) at which female dissections were made.

We performed metagenomic shotgun sequencing on 15 adult females at days 0, 10, and 30 of the first generation (G1), and 60 individuals in the second generation (G2), including 24 nymphs (at days 22 and 34) and 36 adult females (at days 0, 10, and 30) (Fig. 1). An average of 782,400 reads was obtained per sample, and 83% of the reads passed the quality and cleaning filtering (average of 653,290 reads per sample). The analyses were carried out on 35% of the reads that did not represent host contamination (a mean of 226,931 reads per sample). More than 99% of the reads belong to bacteria and archaea that were processed together, principally bacteria. The fungi and viruses represent, on average, 0.03% and 0.015%, respectively. Considering all time points analyzed, 16,194 taxa were identified: 15,703 bacteria, 302 archaea, 146 fungi, and 43 viruses.

### (i) Composition of the gut bacterial community.

The *B. germanica* microbiota is mainly composed of members of phyla *Bacteroidetes* (65%), *Firmicutes* (18%), *Proteobacteria* (15%), and *Fusobacteria* (1.8%) (see Fig. S1 in the supplemental material). The three most abundant genera (*Bacteroides*, *Dysgonomas*, and *Parabacteroides*) belong to the phylum *Bacteroidetes*. The core bacterial microbiota identified from control samples was composed of 120 and 124 genera for adults and nymphs, respectively (see Table S1 in the supplemental material). The nymphal core shares almost all genera included in the adult core, except for five low-abundance genera. Also, the most abundant taxa (>1%) were maintained in both stages.

10.1128/mSystems.01213-20.1 FIG S1 Bacterial composition of the *B. germanica* gut microbiota per sample at the phylum (A) and genus (B) level. The most abundant taxa (>0.5%) are presented. The *y* axis represents the proportion of sequencing reads belonging to each taxon. Download FIG S1, EPS file, 1.1 MB. Copyright © 2021 Domínguez-Santos et al. 2021 Domínguez-Santos et al. https://creativecommons.org/licenses/by/4.0/ This content is distributed under the terms of the Creative Commons Attribution 4.0 International license.

10.1128/mSystems.01213-20.3 TABLE S1 Relative abundance (as a percentage) of the core bacteria in the *B. germanica* gut microbiome. Bacteria included in adult and nymph cooccurrence networks (Fig. 4) are indicated (+). Taxa with a relative abundance over 1% are highlighted. uc, unclassified or unresolved taxa (i.e., at least two or more species or genera from the database match the query reads with similar score). Download Table S1, XLSX file, 0.02 MB. Copyright © 2021 Domínguez-Santos et al. 2021 Domínguez-Santos et al. https://creativecommons.org/licenses/by/4.0/ This content is distributed under the terms of the Creative Commons Attribution 4.0 International license.

### (ii) Diversity and structure of the gut bacterial community.

The comparison of the alpha-diversity (Shannon index and Chao1) and beta-diversity parameters between control adult samples from G1 and G2 gave nonsignificant differences (Wilcoxon test, *P* values > 0.05; ADONIS test, *P* value = 0.6, respectively; see Fig. S2 in the supplemental material). Therefore, Ca and CCa samples were taken as a single adult control group (Ca plus CCa [Ca+CCa]) for further comparisons.

10.1128/mSystems.01213-20.2 FIG S2 Alpha diversity of the *B. germanica* gut microbiome. Shannon index (A) and Chao1 richness estimator (B) of control and experimental samples. Download FIG S2, TIF file, 1.0 MB. Copyright © 2021 Domínguez-Santos et al. 2021 Domínguez-Santos et al. https://creativecommons.org/licenses/by/4.0/ This content is distributed under the terms of the Creative Commons Attribution 4.0 International license.

When we compared the alpha-diversities of control adults and populations treated with kanamycin for one or two generations (Ka and KKa), diversity dropped significantly, mainly in G2 (*P* values < 0.05). After antibiotic removal (KCa and KFa), while the richness (Chao1) reached control levels (*P* values > 0.05), diversity (Shannon index) was significantly lower in KFa (*P* values < 0.05). In nymphs, diversity was also lower after antibiotic treatment in G2 (KKn) (*P* value < 0.05). KCn and KFn richness showed a complete recovery (*P* values > 0.05), whereas diversity remained slightly lower in both cases (*P* values < 0.05). Interestingly, the comparison between nymph and adult controls indicated that nymphs had already acquired the richness and diversity of adults (*P* value > 0.05).

**FIG 2:**
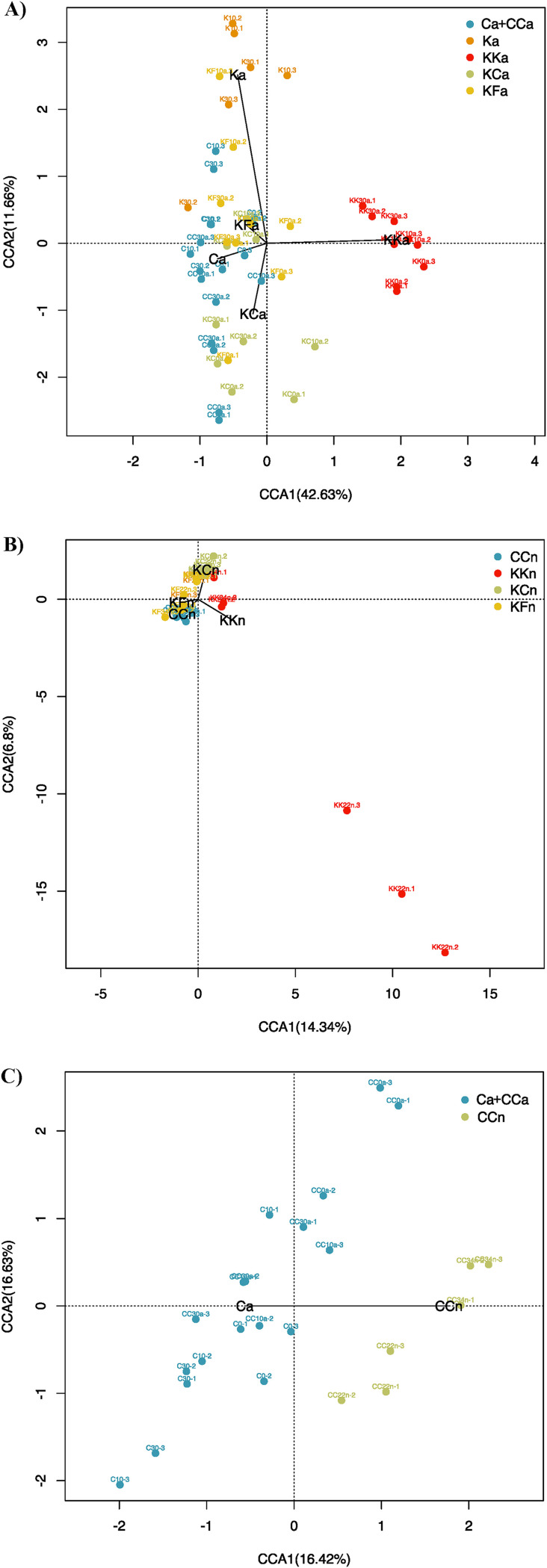
Canonical correspondence analysis (CCA) of *B. germanica* gut microbiome between control and experimental adults (A), control and experimental nymphs (B), and control adults and nymphs (C). The axes represent the percentage of the corresponding total variance explained.

To explore differences in composition between samples, we performed canonical correspondence analyses (CCA; Fig. 2). In adults, control and antibiotic-treated samples had distinct profiles (Fig. 2A). The first axis explained 42.63% of variability, clustering the treated samples at G2 apart from the rest (ADONIS test; *P* value = 0.002). In contrast, KCa (*P* value = 0.08) and KFa (*P* value = 0.07) represent a restored microbiota as the samples grouped with controls.

In nymphs, we observed differences in KKn, mainly because day 22 samples showed an extremely different structure, probably related to its low diversity (*P* value = 0.02) (Fig. 2B). Similar to adults, KCn (*P* value = 0.12) and KFn (*P* value = 0.3) presented a restored microbiota. Again, we found nonsignificant differences between control nymph and adult microbiota composition (Fig. 2C; ADONIS test, *P* value = 0.47).

Control and experimental microbiota composition were compared at the genus level with the ANCOM (analysis of composition of microbiomes) method (Table S2). Thirteen genera were significantly affected in adults after one round of antibiotic treatment (K10a and K30a samples). Most of them were low-abundance taxa, except *Dysgonomonas*, which increased from 13% (controls) to 25% (Ka), and Deltaproteobacteria_uc, which decreased from 5% to 1.37%. In KKa, 64 genera were significantly affected, including those that changed in Ka with the exception of *Dysgonomonas*, which returned to control levels. The most significant changes involved *Bacteroides* (14.4% in control, 45.1% in KKa), *Desulfobrivio* (6.5% and 0.1%), and *Alistipes* (4.3% and 1.03%). All those taxa tend to recover control abundances in KCa and KFa samples. Only 11 and 4 genera were significantly different from controls in KCa and KFa, respectively. Again, most of them were low-abundance taxa (<1%) in control conditions, except Rikenellaceae_uc and *Alistipes* in KCa, and “*Candidatus* Adiutrix” in KFa.

10.1128/mSystems.01213-20.4 TABLE S2 Bacteria with significantly different abundance between control and experimental samples based on the ANCOM test. Upward and downward arrows indicate those taxa that were more and less abundant, respectively, in the specific condition. Download Table S2, XLSX file, 0.01 MB. Copyright © 2021 Domínguez-Santos et al. 2021 Domínguez-Santos et al. https://creativecommons.org/licenses/by/4.0/ This content is distributed under the terms of the Creative Commons Attribution 4.0 International license.

In nymphs, compared with CCn, only seven taxa were significantly affected in the KKn population, while 12 and 2 taxa were significantly affected in the recovered KCn and KFn, respectively. The most affected taxa were present at low abundance except Deltaproteobacteria_uc, Rikenellaceae_uc, and *Fusobacterium* in KCn.

**FIG 3:**
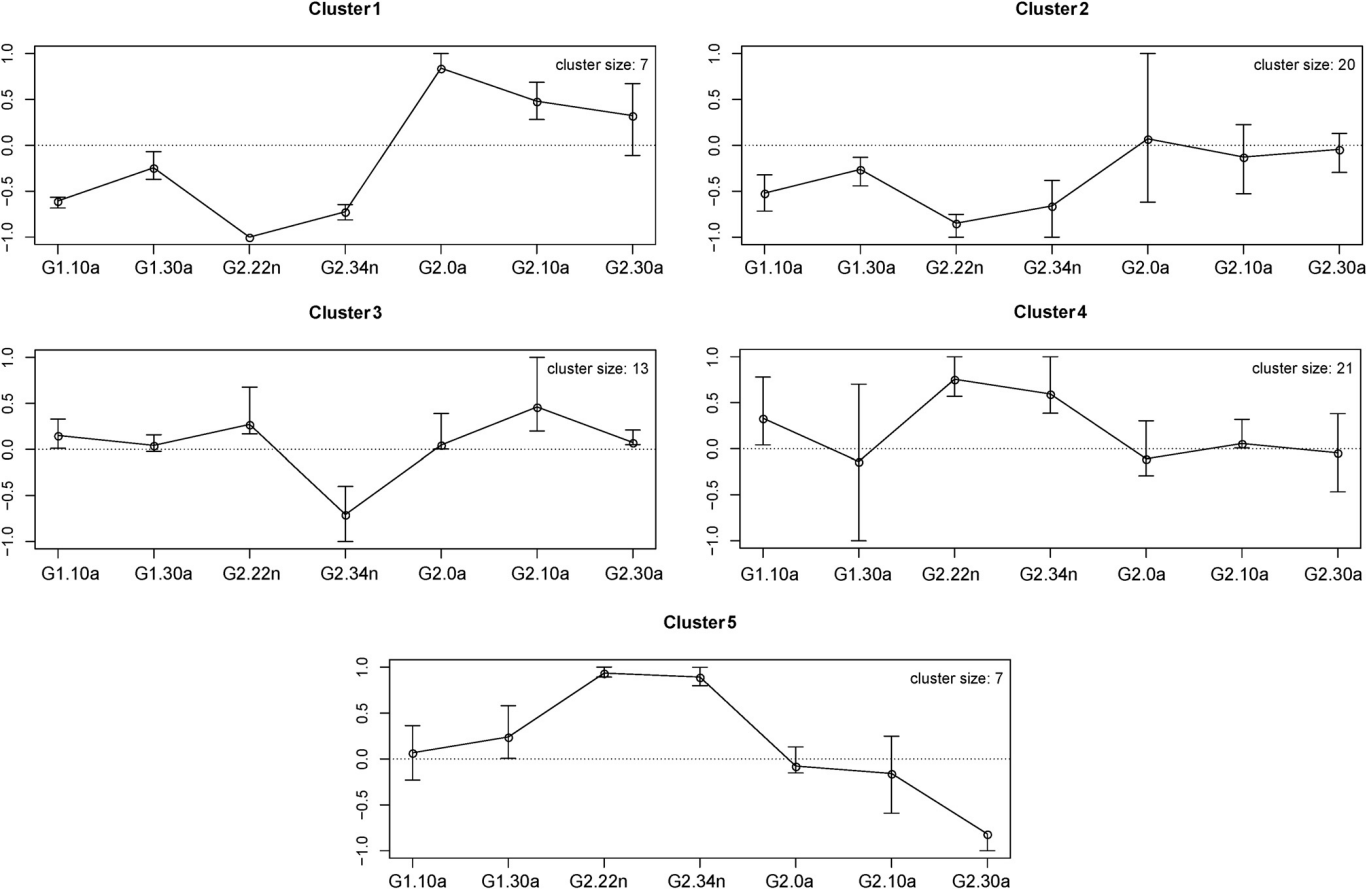
Self-organizing map (SOM) analysis representing those taxa with similar temporal dynamics. Only taxa that were significantly affected by the kanamycin treatment are included. The cluster size indicates the number of taxa in each cluster.

We analyzed the change dynamics along the development for 68 taxa showing statistically significant differences between control and antibiotic-treated samples using a self-organizing map (SOM) approach, which identifies and clusters taxa with a similar relative-abundance pattern over time. We identified five clusters representing different dynamics in response to kanamycin (Fig. 3 and Table S3). Taxa in clusters 1 and 2 showed a similar behavior until G2 nymphal stages, decreasing their abundance. Then, taxa in cluster 1 increased their abundance, while those in cluster 2 recover control values. Cluster 3 showed a tendency to keep or reach control values during development, except for KK34n, with a significant decrease in abundance. Finally, clusters 4 and 5 showed similar behavior in nymphal stages with an increase in resistant taxa. However, whereas adults of G2 return to control levels in cluster 4, taxa belonging to cluster 5 decreased compared to control values.

10.1128/mSystems.01213-20.5 TABLE S3 Cluster affiliation derived from the SOM analysis of bacteria that showed significant differences between control and antibiotic-treated samples in the ANCOM test. Their presence in the adult and nymph cooccurrence networks is indicated (+). Download Table S3, XLSX file, 0.01 MB. Copyright © 2021 Domínguez-Santos et al. 2021 Domínguez-Santos et al. https://creativecommons.org/licenses/by/4.0/ This content is distributed under the terms of the Creative Commons Attribution 4.0 International license.

### (iii) Putative bacterial associations.

**FIG 4:**
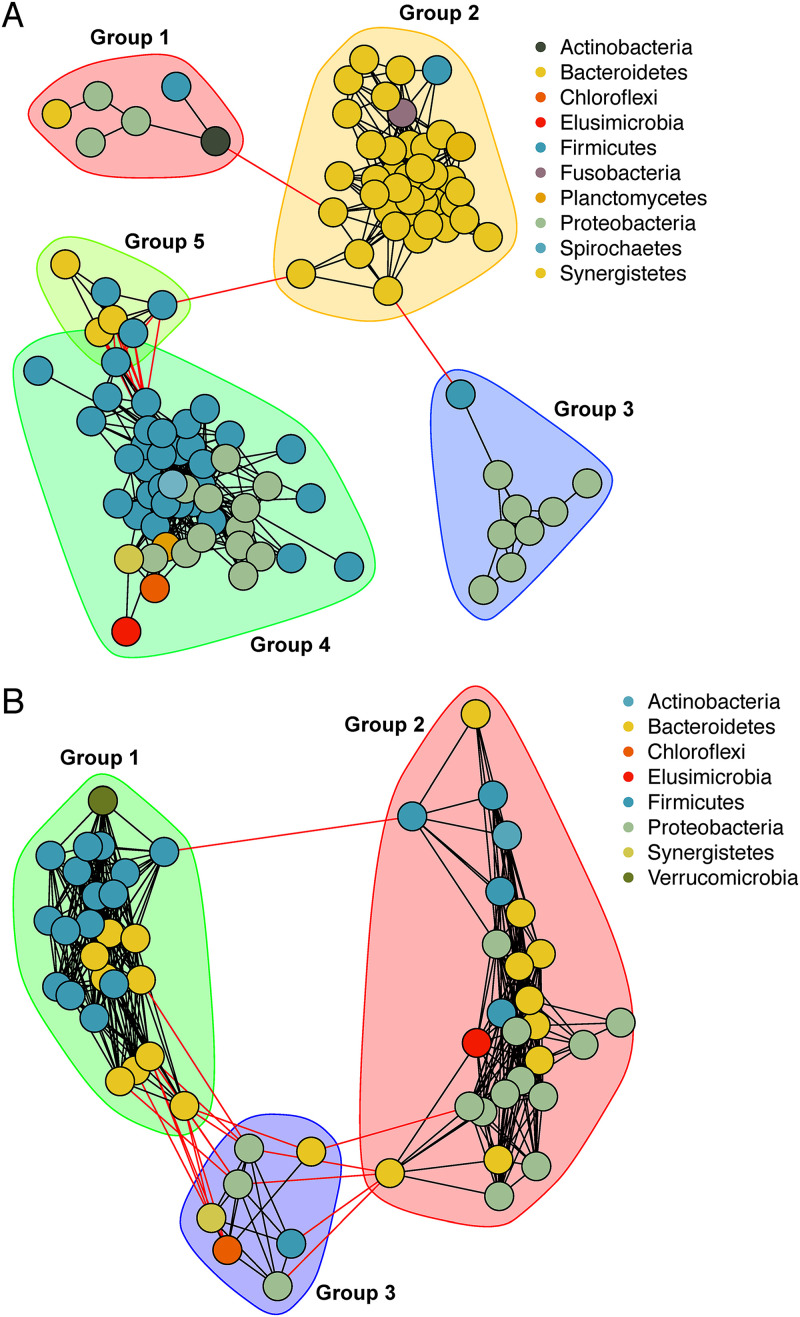
Bacterial cooccurrence networks in the gut microbiota of *B. germanica* in control conditions, considering the genus as the lowest level. (A and B) Adults (Ca+CCa samples) (A) and nymphs (CCn samples) (B). Significant positive associations are shown (*P* value < 0.01). Each node represents a genus, color- coded by phylum. The main clusters of nodes are highlighted. Connections within a cluster are colored red, and connection between clusters are colored black. For a complete list of taxa represented here, see Table S4 in the supplemental material.

To identify potential cooperative relationships between bacterial genera in the gut, we performed a coabundance network analysis for control adults and nymphs (Fig. 4 and Table S4). It will allow us to identify those bacteria that are crucial for the microbial ecosystem's equilibrium and resilience, including the identification of keystone taxa. Like the core microbiota, the networks were enriched in *Bacteroidetes*, *Firmicutes*, and *Proteobacteria*. Most adult network components (105 taxa) were part of this insect’s core bacterial microbiota (Tables S1 and S4). This suggests that they are not only residents but also interact in the gut. The adult network consists of 112 taxa (nodes) clustered in five main groups with a clear phylogenetic signal by phylum (Fig. 4A). This signal by phylum is observed in group 1, mainly dominated by *Bacteroidetes*, in group 2, mostly made up of *Proteobacteria*, and group 4, primarily composed of *Firmicutes* and *Proteobacteria*. The groups with more diversity were group 3, formed by a mix of *Actinobacteria*, *Bacteroidetes*, *Firmicutes*, and *Proteobacteria*, and group 5, containing *Bacteroidetes* and *Firmicutes*. In networks, highly connected nodes and articulation points (nodes that, when absent, disconnect the network) are generally considered “keystone” taxa. We identified 10 bacterial taxa as highly connected nodes and 12 as articulation points (Table S4).

10.1128/mSystems.01213-20.6 TABLE S4 Gut microbiome bacteria that constitute the bacterial network in control adult and nymphal stages (Fig. 4). Keystones are indicated with asterisks, highly connected taxa are shown in black, and articulation points are shown in red. Taxa absent in the control core are written in red. Download Table S4, XLSX file, 0.02 MB. Copyright © 2021 Domínguez-Santos et al. 2021 Domínguez-Santos et al. https://creativecommons.org/licenses/by/4.0/ This content is distributed under the terms of the Creative Commons Attribution 4.0 International license.

The control nymph network is smaller than the adult network, with three groups and 58 nodes, 55 of them part of the core microbiota (Fig. 4B and Table S4). The phylum distribution was similar to adults, and almost all taxa present in the nymph-based network were also present in adults. We did not find articulation points, but 10 taxa were highly connected nodes.

### Nonbacterial components of the *B. germanica* microbiome.

For the first time in this species, we analyzed the composition of the nonbacterial fraction (archaea, fungi, and viruses) of its gut microbiome. We grouped the samples (adults and nymphs) into three categories: control conditions (C+CC), kanamycin-treated (K+KK), and recovered after antibiotic treatment (KC+KF).

**FIG 5:**
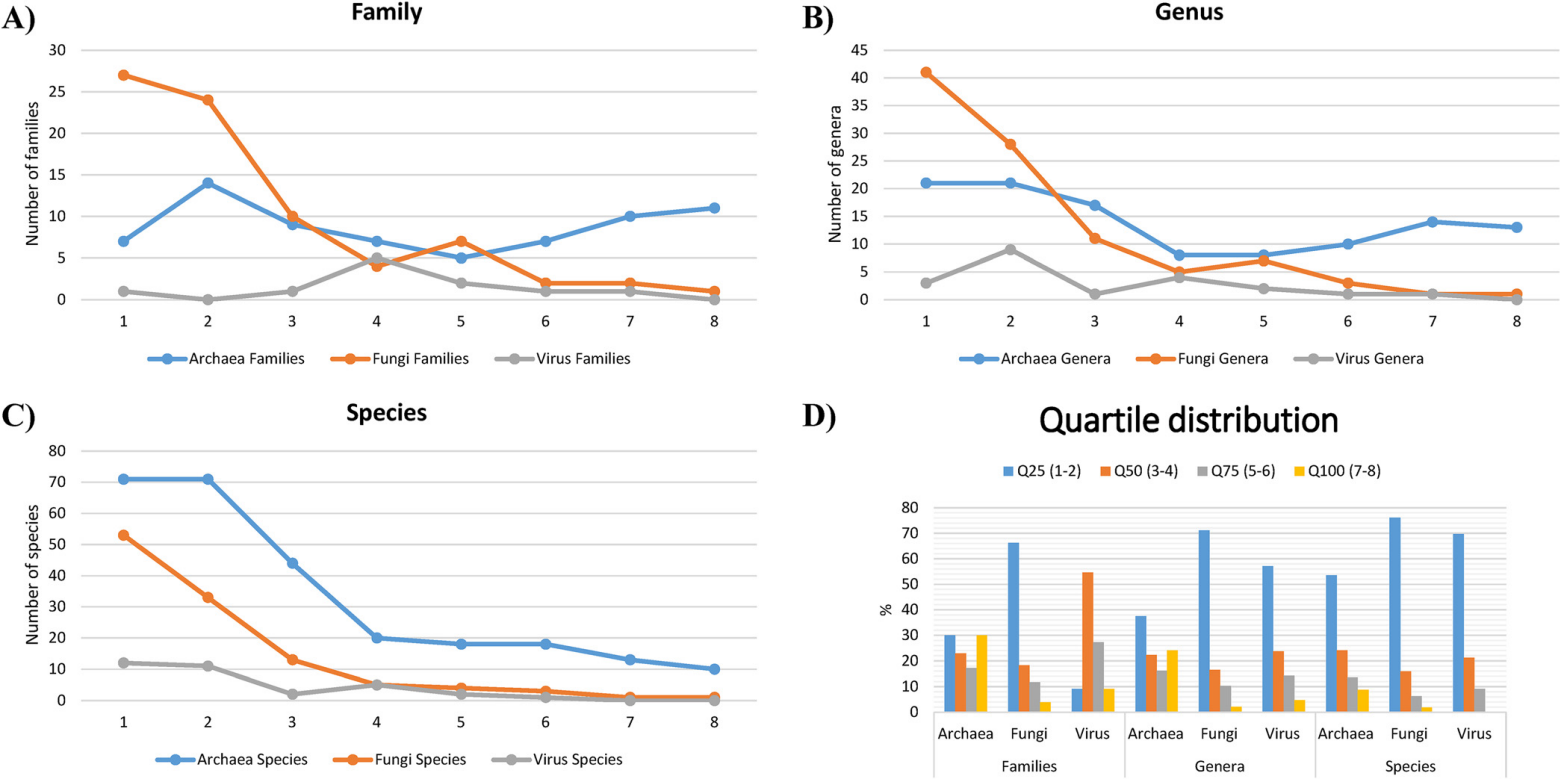
Summary of archaea, fungi, and viruses identified in the microbiome of *B. germanica.* (A to D) Number of archaeal, fungal, and viral families (A), genera (B), and species (C) identified in the control samples at the eight time points and their global quartile distribution (D).

In controls, in the archaeal fraction, we identified 265 species from 112 genera belonging to 70 families (Fig. 5 and Table S5). The most widespread archaea, present in more than three-quarters of the sampling times (Q100), belong to 21 out of the 70 families identified. This number was the same for the recovered group at G2, and slightly higher than the antibiotic-treated samples (64 families), indicating that diversity was not drastically affected by kanamycin. Control and recovered samples were dominated by *Methanosarcinaceae* (22.53%), *Methanobacteriaceae* (12.13%), and *Methanomassiliicoccaceae* (6.34%) (see Data Set S1.1 in the supplemental material). In antibiotic-treated samples, these three families were among the most abundant, but with the nonclassified *Euryarchaeota* in third position (5.91%).

10.1128/mSystems.01213-20.7 TABLE S5 Archaea, fungi, and viruses detected in control gut samples of *B. germanica*. (A) Families and species sorted by quartile; (B) distribution of taxa, measured as relative abundance at family and species level. N, presence in number of samples. Color indicates the quartile: blue, Q25; red, Q50; green, Q75; purple, Q100. uc, unclassified or unresolved taxa (i.e., at least two or more species or genera from the database match the query reads with similar score); na, not assigned (i.e., taxa lacking annotation in the taxonomy database, because no official name is accepted yet for them). Download Table S5, XLSX file, 0.02 MB. Copyright © 2021 Domínguez-Santos et al. 2021 Domínguez-Santos et al. https://creativecommons.org/licenses/by/4.0/ This content is distributed under the terms of the Creative Commons Attribution 4.0 International license.

10.1128/mSystems.01213-20.10 DATA SET S1 Relative abundance of archaea, fungi, and viruses detected in the gut microbiome of *B. germanica*. The three replicates per sample appear aggregated. The color scale ranges from white (absence) to dark green (most abundant). (A) Relative abundance in control samples at the family and species level. (B) Relative abundance in experimental samples at the family level. (C) Relative abundance expressed as a percentage in both control and experimental samples at the family level. Taxa are sorted by average global abundance (A and B) or alphabetically (C). (D) Barplot of the data presented in panel C. Colors preceding the family names in panel C are those used in the barplot. uc, unclassified or unresolved taxa; na, not assigned. Download Data Set S1, XLSX file, 0.3 MB. Copyright © 2021 Domínguez-Santos et al. 2021 Domínguez-Santos et al. https://creativecommons.org/licenses/by/4.0/ This content is distributed under the terms of the Creative Commons Attribution 4.0 International license.

The mycobiota comprised 77 families, 97 genera, and 113 species (Fig. 5 and Table S5). Three families (Basidiobolaceae, Neocallimastigaceae, and Aspergillaceae) belong to the Q100 category. The number of families identified in the controls (*n* = 77) was slightly higher than in recovered samples (*n* = 73), and especially compared to the kanamycin-treated samples (*n* = 66). The most abundant families in control and recovered samples (Data Set S1.3) were Neocallimastigaceae (10.58%), Saccharomycetaceae (7.63%), and Aspergillaceae (5.71%), accounting for less than a quarter of fungal reads. However, in antibiotic-treated samples, Saccharomycetaceae (13.90%) was the most abundant, followed by Malasseziaceae (11.12%) and uncharacterized Exobasidiomycetes (8.68%).

Viral reads were scarce and, therefore, more limitedly distributed through the samples than other microbiome components. We found viruses from 11 families, involving 33 species from 21 genera in control conditions (Fig. 5 and Table S5). In recovered and kanamycin-treated samples, 11 and 6 families were identified, respectively. Only reads from one bacteriophage family, *Siphoviridae*, were detected in seven out of eight control sampling times. This family was the most abundant in controls (28.85%) (Data Set S1.5), followed by *Myoviridae* (11.00%) and *Podoviridae* (4.87%). In kanamycin-treated samples, the relevance of nonassigned viruses was even higher (38.29%), followed by *Phasmaviridae* (28.37%), due to its particular abundance in nymphs from day 22 (84.62%). However, the presence of *Myoviridae* (12.27%) and *Siphoviridae* (11.17%) was comparatively reduced in this group, maybe reflecting the loss of bacterial hosts in antibiotic-treated cockroaches.

### Variation of archaea, fungi, and viruses in development.

We compared the archaea, fungi, and viruses found in nymphs and adults to evaluate whether there was a significant shift in those communities during development (Table S6). In both stages, the most abundant archaeal families were *Methanosarcinaceae*, *Methanobacteriaceae*, and *Methanomassiliicoccaceae*. The smaller number of samples could partially explain the decrease in the number of families identified in nymphs. The lack of detection was even more pronounced regarding fungi. In this case, the most abundant families in both stages were Neocallimastigaceae and Saccharomycetaceae. As for viruses, 8 out of 11 families found in adults were detected in nymphs. Bacteriophage families *Myoviridae* and *Siphoviridae* were the most abundant in nymphs, similar to adults.

10.1128/mSystems.01213-20.8 TABLE S6 Relative abundance of archaea, fungi, and viruses in control samples at the family level from samples at nymphal and adult stages. Families are sorted by average global abundance. The The color scale ranges from white (absence) to dark green (most abundant). uc, unclassified or unresolved taxa; na, not assigned. Download Table S6, XLSX file, 0.01 MB. Copyright © 2021 Domínguez-Santos et al. 2021 Domínguez-Santos et al. https://creativecommons.org/licenses/by/4.0/ This content is distributed under the terms of the Creative Commons Attribution 4.0 International license.

### Functional analyses of the *B. germanica* gut microbiome.

For all samples, functional profiles were predicted based on the KEGG database. Similar to the taxonomic analysis, Ca and CCa showed no significant differences in gene profiles (ADONIS test, *P* value = 0.56), and we joined them as a single adult control group. We analyzed how the functional profile was affected by the antibiotic using the ANCOM test at the KEGG pathway level (Table S7). Compared to controls, only 10 pathways changed after one generation of treatment. However, in the second generation of treatment, the antibiotic strongly altered the microbiome functionality, with significant changes in 51 pathways, including carbohydrate and lipid metabolism, transporters, two-component system, bacterial toxins, and antimicrobial resistance genes. As for the populations not treated in G2, most pathways recovered.

10.1128/mSystems.01213-20.9 TABLE S7 KEGG pathways with significantly different abundance between control and experimental samples based on the ANCOM test. The comparisons and a summary of the results are presented. Upward and downward arrows indicate those functions that were more and less abundant, respectively, in the specific condition. Download Table S7, XLSX file, 0.01 MB. Copyright © 2021 Domínguez-Santos et al. 2021 Domínguez-Santos et al. https://creativecommons.org/licenses/by/4.0/ This content is distributed under the terms of the Creative Commons Attribution 4.0 International license.

Control adults and nymphs did not have a distinct functional profile (ADONIS test, *P* value = 0.56). In nymphs, two generations of antibiotic treatment affected only one pathway related to bacterial invasion of epithelial cells. However, in recovered populations, KCn showed 19 pathways with significant differences compared to controls, while KFn functions were restored, as in KFa (Table S7).

### Gut microbiome-associated resistome of *B. germanica*.

We analyzed the ARG profile of 188 *B. germanica* gut metagenomes: 75 from this kanamycin study plus 113 from a previous study in which ampicillin and vancomycin were used to disrupt the insect microbiota (13). Altogether, we analyzed 36 control samples, 65 samples from different antibiotic treatments (ampicillin, 22; vancomycin, 22; kanamycin, 21), and 87 samples from populations restored after one generation of each treatment (27, 30, and 30 samples, respectively). About 0.1% of reads per sample were positive against FARME DB entries, and 625 described protein-coding genes were identified.

**FIG 6:**
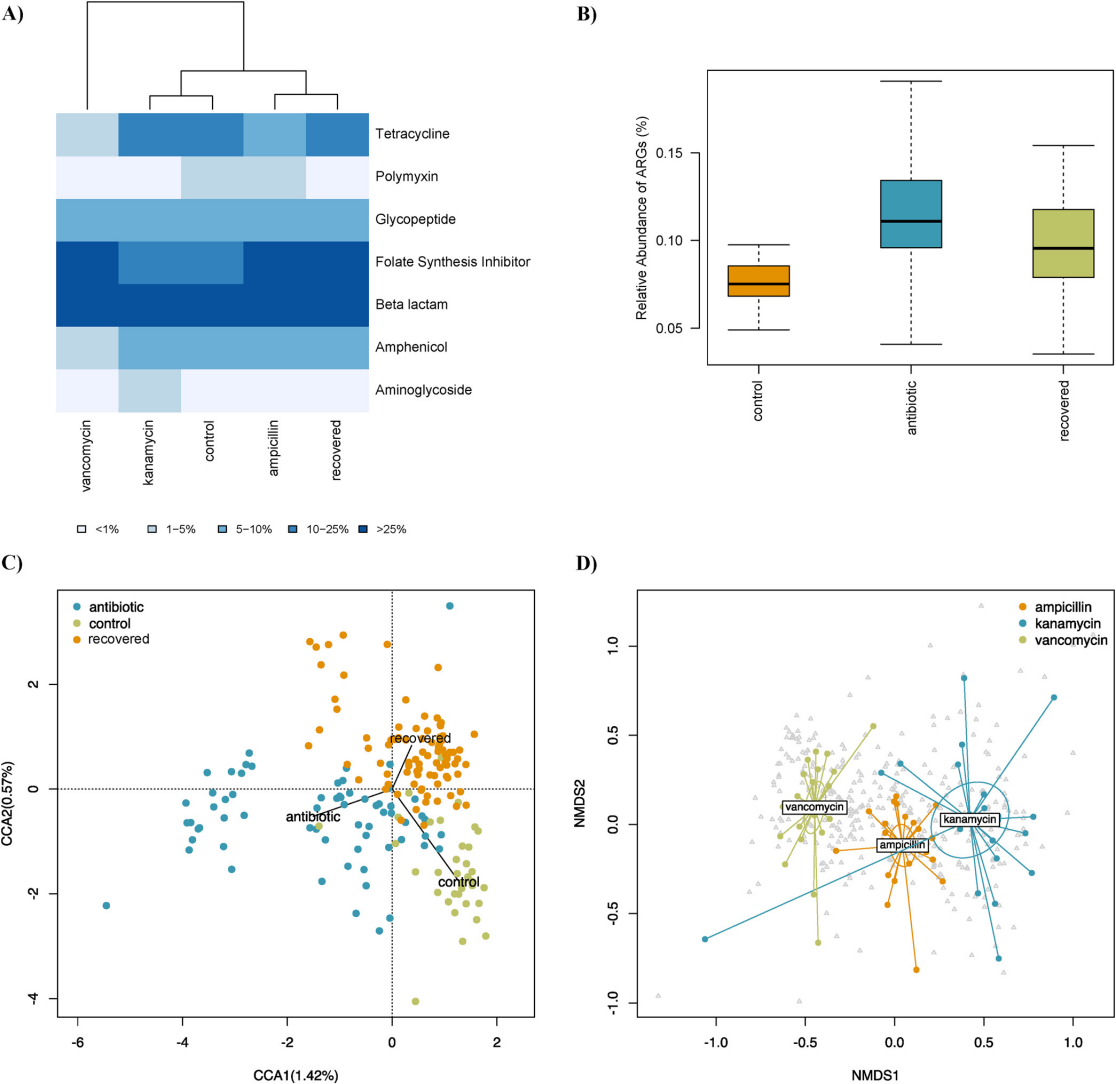
Resistome analyses of *B. germanica* gut microbiome on samples from control, antibiotic-treated (with ampicillin, vancomycin or kanamycin), and recovered populations. (A) Heatmap and clustering are based on identified ARGs grouped by resistance type. Heatmap colors show the percentage range of sequences assigned to the taxa. (B) Relative abundance of ARGs in the analyzed metagenomes. (C) CCA of the resistome composition. (D) Nonmetric multidimensional scaling (NDMS) analysis of the resistome composition among the different antibiotics used. *P* values of the ADONIS test comparisons: ampicillin versus vancomycin, 0.001; ampicillin versus kanamycin, 0.001; kanamycin versus vancomycin, 0.001.

Untreated samples were taken to reference the natural ARG reservoir in cockroach populations. In these control samples, we identified 325 protein models. Most ARGs conferred resistance against beta-lactams (51%), folate synthesis inhibitors (20%), tetracyclines (11%), amphenicols (8%), glycopeptides (7%), polymyxins (1%), and aminoglycosides (0.4%) (Fig. 6A). Some macrolide and sulfonamide ARGs were also present. Besides, around 20 different mobile element-related components were identified, including genes coding for transposases, conjugative DNA transfer proteins associated with type IV secretory systems, plasmid recombination enzymes, phage integrases, and recombinases. These findings suggest a capacity to mobilize DNA, including ARGs.

We found a higher relative abundance of ARGs in antibiotic-treated samples than in control samples (0.12% versus 0.08%; *P* value = 4.348e−09) (Fig. 6B). Interestingly, samples recovered after one generation of antibiotic treatment also had an ARG abundance (0.1%) higher than control samples (*P* value = 6.204e−05).

The three groups (control, antibiotic treated, and recovered) had different ARG composition and abundance profiles, although considerable variability was observed (Fig. 6C). Control and antibiotic-treated samples showed significant differences (ADONIS test, *P* value = 0.001). Recovered groups presented differences with control and antibiotic-treated clusters (*P* values = 0.002 and 0.001, respectively). Some of the resistome differences after antibiotic cessation compared to control were a higher relative abundance of folate synthesis inhibitors and a lower abundance of genes involved in resistance to beta-lactams and polymyxins (Fig. 6A).

The three analyzed antibiotics shaped the resistome in a specific way (Fig. 6D). Regarding the most common resistances identified in control samples, we detected distributions significantly different in treated samples (with ampicillin, kanamycin, and vancomycin, respectively; *P* values < 0.05 except when indicated) (Fig. 6A) as follows: beta-lactams (46%, 53%, and 26%), folate synthesis inhibitors (32%, 16%, and 63%), tetracyclines (6%, 13%, and 1%), amphenicols (7%, 9%, and 3%; *P* values < 0.05 except ampicillin versus kanamycin), glycopeptides (8%, 8%, and 6%; *P* values > 0.05), and aminoglycosides (0.2%, 1%, and 0%; *P* values > 0.05 except kanamycin versus vancomycin).

### Effects of kanamycin on the *Blattabacterium* population of *B. germanica*.

To quantify the amount of *Blattabacterium* present in the fat body of control and kanamycin-treated insects, we quantified *ureC* with real-time quantitative PCR (qPCR) (data not shown). We did not find statistically significant differences in the average copy number of *ureC* per nanogram of fat body DNA in any comparison (Wilcoxon test, *P* values > 0.05). The quantification of the average number of copies of the host control gene *actin5C* did not show significant changes between G1 and G2 (*P* value = 0.72). These results indicate that the *Blattabacterium* population is not affected by kanamycin.

## DISCUSSION

The present work aimed to investigate the role of the gut microbiota in *B. germanica*, as well as to disentangle its assembly and variation from nymph hatching to adult life by perturbing a control population with kanamycin (an aminoglycoside, inhibitors of protein synthesis that act against Gram-negative bacteria and some Gram-positive bacteria). In previous studies by our group, three antibiotics were tested, rifampicin (a broad-spectrum antibiotic) (49), vancomycin (a glycopeptide acting against Gram-positive bacteria), and ampicillin (a beta-lactam acting against Gram-positive and some Gram-negative bacteria) (13). In this work, we did not find a significant effect of the kanamycin treatment on the endosymbiont *Blattabacterium* despite being Gram negative. This can be due to the molecular structure of kanamycin. Aminoglycosides cannot be properly absorbed by the intestinal mucosa (55). Then, kanamycin might be unable to reach the fat body in which the bacteriocytes hosting *Blattabacterium* are located and could not attack the endosymbiont even when it is out of the bacteriocytes for its vertical transmission. Another possibility is that the dose used is too low to affect the endosymbiont. In any case, this result indicates that any variation detected in the gut microbiome will not be caused by a fitness change in the cockroach due to its essential endosymbiont loss. It is worth mentioning that *Blattabacterium* was also not affected by ampicillin (13). Thus, at present, rifampicin is the only tested antibiotic that acts against the endosymbiont when it leaves the bacteriocyte to invade the oocytes (49).

The abundance of the main bacterial phyla in the gut microbiota of *B. germanica* was the same for nymphs and adults in control populations (see Fig. S1 and Table S1 in the supplemental material). Their bacterial community is mostly composed of *Bacteroidetes*, *Firmicutes*, *Proteobacteria*, and *Fusobacteria*. These results agree with those previously obtained for adults by our group (13, 49), leading to the determination of the core bacterial phyla of this species, at least under laboratory conditions. Among the most abundant core genera, we found *Bacteroides* (*Bacteroidetes*), *Desulfovibrio* (*Proteobacteria*), *Clostridium* (*Firmicutes*), and *Fusobacterium* (*Fusobacteria*). All of them have been identified in previous *B. germanica* gut microbiota studies and participate in polysaccharide and protein digestion, nitrogen fixation, and protection against pathogens (13, 48, 49).

When exploring taxon associations through network analysis, we found that core bacteria were involved in putative ecological interactions in adults and nymphs (Fig. 4 and Table S4). It suggests that they are not only residents of the gut microbiome but also provide stability to the whole microbial ecosystem. In fact, we identified some keystone species, which deserve further research to clarify their role in the equilibrium of the gut microbiome. The network groups showed a phylogenetic signal as they tend to contain bacteria belonging mainly to one phylum, a trend that has also been found in the human bacterial microbiome (56, 57). The cooccurrence of closely related microorganisms indicates that cooperation predominates over competition in these taxonomic groups. Interestingly, bacterial networks were different in adults and nymphs. However, the differences might reflect the fact that the nymph’s network is smaller, as it seems to be a subset of the adult one where other genera have replaced the keystone taxa except for *Bacteroides* and Pseudomonas, which seem to be essential during the whole life, although the latter is not highly abundant. This finding highlights the crucial role of minority species in microbial ecosystems. As in previous studies, it seemed that, under stable conditions, the microbiota acquired after hatching is assembled in a defined way determined very early in development (47). However, our new results indicate that the interactions that structure the community had not yet been well established before reaching the adult stage. Nevertheless, some not especially abundant but essential components are already incorporated into the gut microbiota in early life.

The essentiality of *Bacteroides* and Pseudomonas can also be deduced from the SOM analysis results (Fig. 3). These two taxa (belonging to clusters 3 and 1, respectively) showed an opposite profile during antibiotic treatment, but when treatment ceased in G2, their relative abundance stabilizes in adults, returning to control levels. As in many animal microbiotas, *Bacteroides* could participate in polysaccharide digestion and energy metabolism and protect from pathogens by stimulating the immune system due to the production of polysaccharides or antibacterial peptides (58). Pseudomonas could also participate in food digestion since amylolytic, cellulolytic, xylanolytic, lipolytic, and esterase activities from these bacteria have been reported in some insects (59, 60).

As expected, kanamycin exposure produced a drastic shift in taxonomical composition (Fig. 2 and Table S2). Although the effects were, in general, mild in the first generation of treatment, in which few taxa were affected, they were more evident in the second generation, indicating a progressive increase of resistant taxa and the corresponding decrease of sensitive taxa. This could be related to a buffering effect caused by other coexisting bacteria in the first antibiotic pulse since the treatment was initiated in adults, when the ecological network was already assembled. The same effect was also observed in experiments using rifampicin (49). *Fusobacterium* and *Desulfovibrio* were the most affected genera and virtually disappeared after two generations of kanamycin treatment. Species of both genera have been proposed to have nutritional functions (48, 61, 62). On the other hand, the genus *Bacteroides*, inherently resistant to aminoglycosides (63, 64), increases its abundance. This suggests that resistant species can easily occupy the niches left open by those sensitive to kanamycin. Furthermore, they can provide lost nutritional functions due to their ability to digest complex sugars and polysaccharides for growth (65). Further studies, including bacterial biomass quantification, will confirm whether the relative abundance results reflect real absolute abundance changes.

The two untreated populations in G2 (KC and KF) showed a trend to recovery, slightly faster with added feces to the diet, as it was previously shown (13, 49). We also detected that 34-day-old nymphs had already been colonized by the main taxa that constitute the stable adult microbiota. In recovered populations, nymphs whose diets had been supplemented with feces had the same microbiota composition as controls (Fig. 2). Therefore, regarding resilience in this species’ microbiota, coprophagy is an advantageous strategy for the offspring, as previously postulated (66–69).

In parallel to taxonomic changes, the second generation of cockroaches treated with kanamycin strongly disturbed the microbiome functionality (Table S7), with several metabolic routes altered. Interestingly, many KEGG pathways that increased after treatment were involved in antibiotic resistance, defense systems, and toxin production, suggesting an immediate expansion of opportunistic and antimicrobial-resistant bacteria.

The resistome analysis confirmed the presence of multiple ARGs in the gut metagenomes of *B. germanica* (Fig. 6). Those genes confer resistance against antibiotics used in clinics, agriculture, and farming, such as beta-lactams, folate synthesis inhibitors, tetracyclines, amphenicols, glycopeptides, polymyxins, or aminoglycosides. Reservoirs of ARGs have also been identified in other gut microbiomes and environmental metagenomes (52, 54, 70, 71). A notable result in our model is the significant increase in the relative abundance of ARGs after different antibiotic treatments, generating profiles associated with the antibiotic type used (Fig. 6B and C). The ARGs increase in an antibiotic-specific manner has been described in other gut microbiomes under different antimicrobial therapies, including humans (72, 73). Moreover, after cessation of the antibiotic selection pressure, the resistome retained a high relative abundance of ARGs, indicating that even a single course of antibiotic treatment might permanently increase the presence of those genes at least for two generations. Nevertheless, it is important to notice that *B. germanica* control non-antibiotic-treated populations present ARGs that remain in the absence of direct selection, as occurs in mosquitoes (71). Therefore, ARGs seem to be circulating on this cockroach microbiome in the natural environment.

Beyond ARGs, we identified more than 20 different genetic elements involved in DNA mobilization, which can participate in resistance transmission. Especially, we found transposases (mainly belonging to the DDE family) that form part of DNA transposons that are frequent carriers of antibiotic resistance (74). Therefore, the gut microbiome not only acts as a reservoir of antibiotic resistances but can also mobilize them. Cockroaches are widespread and live in proximity to humans. Thus, similar to other insects (71), they might represent a public health problem due to their role as potential ARG transmission vectors.

Most gut microbiome studies focus on bacteria, while archaea, fungi, protozoa, and viruses remained mostly unexplored. We detected 70 families of archaea (Table S5), suggesting a relevant role of these microorganisms on host physiology as occurs in other animals (34). The most abundant species in adults and nymphs correspond to families *Methanosarcinaceae*, *Methanobacteriaceae*, and *Methanomassiliicoccaceae*. Many cockroach species carry methanogenic archaea in their hindguts (75), sometimes as endosymbionts of anaerobic ciliated protozoa that occupy the same gut compartment (76, 77). In addition to their role in hydrogen transfer, they might contribute to the hindgut nitrogen-carbon balance by nitrogen fixation (78). Most archaea have a broad-spectrum resistance to antibiotics due to their structural and biochemical characteristics (79). In accordance with this fact, we did not detect a significant decrease in their abundance caused by kanamycin (see Data Set S1 in the supplemental material).

Unlike archaea, the observed distribution of fungal and viral taxa indicates that their representatives were not consistently reported across samples (Table S5 and Data Set S1). Therefore, our data cannot define a fungal and viral core microbiome for the gut of *B. germanica*. More samples and deeper sequencing would be required to overcome the scarcity of these reads compared to bacterial ones. Nevertheless, a closer look at the fungal community reveals that Ascomycota, Basidiomycota, and Zoopagomycota were the dominant phyla in the mycobiota of *B. germanica*. Ascomycota and Basidiomycota are also the most abundant fungi in the guts of other insects (80–82). Regarding viruses, the not assigned reads were very abundant, and no viral family was present in more than three-quarters of samples. Only bacteriophages of the family *Siphoviridae* were abundant in controls, whereas *Phasmaviridae* were more abundant in antibiotic-treated populations. Interestingly, the latter is a family of negative-sense RNA viruses known to infect insects. The *Wuchang cockroach orthophasmavirus 1*, belonging to this family, is integrated into its host genome (83). As for bacteriophages, their presence in the gut microbiome can control the bacterial population through cell lysis, thus influencing bacterial diversity and metabolism (84) and facilitating horizontal gene transfer.

### Conclusions.

Antibiotics are a useful tool to experiment with animal-microbiota systems. Different studies carried out by our group in *B. germanica* revealed a hindgut core bacterial microbiome that is altered by antibiotic treatment but is quickly recovered after treatment cessation. The core bacterial microbiome is acquired early in the cockroach life, during nymphal stages, although it is not entirely stabilized until they reach the adult stage, when the cooperative networks are well defined to contribute to the insect metabolism. We could not determine a core archaeome, mycobiome, and virome for this species, and further studies targeting these fractions would be necessary to better understand these communities. A significant finding of our study is the identification of cockroaches as natural reservoirs of ARGs, which can increase in response to antibiotic treatments and be mobilized favoring the increase of antibiotic-resistant microorganisms. Because these insects live in close association with humans, this can cause a biomedical problem that needs to be considered.

## MATERIALS AND METHODS

### Blattella germanica rearing conditions.

A population of B. germanica originating from a laboratory population housed by X. Bellés’ group at the Institute of Evolutionary Biology (CSIC-UPF, Barcelona, Spain) was reared in climatic chambers at the Cavanilles Institute of Biodiversity and Evolutionary Biology (University of Valencia) in plastic jars with aeration. The environment in which the cockroaches lived was controlled and maintained constant during the experiments: photoperiod of 12 h of light and 12 h of darkness, 26°C, and 65% humidity. The populations were fed dog food pellets (Teklad global 21% protein dog diet 2021C; Envigo, Madison, WI, USA), and water was supplied *ad libitum.* The antibiotic kanamycin (Thermo Fisher Scientific, Carlsbad, CA, USA), an aminoglycoside acting mainly against Gram-negative and some Gram-positive bacteria, was supplied with water at 0.2 mg/ml.

### Experimental design.

A synchronized adult population composed of individuals collected between 0 and 48 h after ecdysis (considered adults at time zero) started the first generation (G1) (see Fig. 1 for a scheme of the experimental design and a summary of all samples analyzed). Before any treatment, three female cockroaches were dissected, and their hindgut and fat body were collected (C0a samples). Then, the population was divided into two sex balanced adult subpopulations: one, consisting of 36 males and 36 females, was not treated with antibiotics and served as a control population (C), whereas the other, consisting of 30 males and 30 females, was treated with kanamycin 0.2 mg/ml (K population). At 10 and 30 days (10a and 30a) after the adult population’s establishment, three females were dissected and processed like the C0a samples. Just before nymphs could hatch (i.e., when the oothecae were fully mature), the K population was divided into two groups to generate the second-generation (G2) populations with or without antibiotic. This step was performed to ensure that the newly born nymphs that will belong to the non-antibiotic-treated G2 groups have never had direct contact with kanamycin. Newly hatched nymphs from the group maintained on kanamycin initiated the antibiotic-treated population (KK). The nymphs hatched in the kanamycin-free environment were then separated into two new populations: one fed only control diet without any further treatment, giving rise to the KC population, while the second one was supplemented with feces obtained from the C population, giving rise to the KF population. As a G2 control, a fourth population (CC) was established from the C population. In order to analyze samples throughout the cockroach life cycle in the four G2 populations, both nymph and adult samples were taken at several time points. Nymphs were taken at 22 and 34 days (22n and 34n), and female adults at 0, 10, and 30 days old (0a, 10a, and 30a). We dissected three specimens per condition and collected their hindguts and fat bodies. The fat body could not be collected from nymphs of day 22 due to its scarceness. Overall, 25 time points and 75 individuals were dissected throughout the two generations (15 in G1 and 60 in G2). Throughout the text, the K, KK, KC, and KF are grouped under the name of experimental populations, in opposition to control ones (C and CC).

### Cockroach dissection.

Cockroaches were anesthetized using a CO_2_ stream and dissected under a stereomicroscope to collect the fat body tissue and hindgut. After the gonads were removed, the fat body was collected in Krebs-Ringer bicarbonate buffer (Sigma-Aldrich, St. Louis, USA), frozen in liquid nitrogen, and stored at −80°C. The hindgut was opened, cleaned in Krebs-Ringer buffer to remove fecal residues, and stored at −80°C as well.

### DNA extraction, quantitative PCR, and sequencing.

DNA extraction from the fat body and hindgut was carried out as previously described (13), and the total DNA was quantified with a Qubit 3.0 fluorometer (Thermo Fisher Scientific, Carlsbad, CA, USA).

Quantification of the *Blattabacterium* population was done on fat body samples by measuring the number of copies of *ureC* (GenBank accession number NC_013454.1 [40]), exclusively present in the endosymbiont. The quantification of host *actin*5*C* (GenBank accession number AJ862721.1 [85]) was used as an internal control. The primers used were previously described by Rosas et al. (49). Quantitative PCR was done using an ArialMX real-time PCR system (Agilent Technologies, Germany), and the number of copies was normalized per nanogram of DNA extracted from the fat body. Wilcoxon test (adjusted *P* value by false discovery rate [FDR] method) was applied to analyze statistical differences.

One nanogram of each sample (0.2 ng/μl) from the hindgut was used for shotgun library preparation for high-throughput sequencing using the Nextera XT DNA sample preparation kit (Illumina, Inc., San Diego, CA, USA) according to the manufacturer’s protocol. Sequencing was carried out using the Illumina MiSeq (2 × 300 bp) technology at the Sequencing and Bioinformatic Service facilities of the FISABIO (Foundation for the Promotion of Health and Biomedical Research of Valencia Region, Spain). All 75 samples were sequenced in the same MiSeq run, and three biological replicates were analyzed. The total DNA extracted from the fat body was used for qPCR analyses.

### Bioinformatics methods: quality control, taxonomic and functional analyses.

Metagenomics analyses were performed on three biological replicates per time point for each purified hindgut microbiome DNA sample.

Quality control was performed using PRINSEQ-lite v0.20.4 (86), which filters the reads to remove sequence copies, short or long sequences, or low-quality ones, and the FastQC tool for a detailed quality control report of the reads. Forward and reverse reads passing the quality check were joined using FLASH v1.2.11 (87), applying the following parameters: min-overlap 10, max-overlap 150, and max-mismatch-density 0.1. Next, reads were mapped against the *B. germanica* reference genome (88) using Bowtie2 v2.3.4.1 (89) with end-to-end and sensitive options.

### (i) Taxonomic and functional annotation of the bacterial fraction.

Taxonomic annotation was performed by Kaiju v1.6.2 (90) on the cockroach-free reads. DNA sequences were translated into all reading frames, and maximum exact matches (MEMs) were searched across the protein NCBI BLAST nr+euk database for bacteria, archaea, fungi, and viruses. The LCA (lowest common ancestor) algorithm was used to phylogenetically classify the taxa and create a taxonomical abundance matrix, using the package R (91). Each sample was normalized to the relative abundance to avoid the bias associated with sequencing depth. Only taxa with a relative abundance of >0.1% per experimental group were included in the taxonomic abundance matrix.

Assembly of the *B. germanica*-free sequences was performed by MEGAHIT (92). Contigs were scanned for open reading frames (ORFs) via Prodigal (prokaryotic dynamic programming gene-finding algorithm [93]). Functional annotation of ORFs was done via HMMER alignments (94) against KEGG hidden Markov models (HMM [95]).

### (ii) Analysis of the communities of archaea, fungi, and viruses.

For the descriptive analysis of the archaea, fungi, and viruses inhabiting the hindgut of *B. germanica*, the matching hits from these three groups were extracted from the previously generated taxonomic abundance matrix. Nonaggregated sample composition was calculated at family, genus, and species level, and taxa were sorted according to the number of samples where they could be identified, to establish which ones were shared by several samples or only occasionally found. In this regard, quartiles were set up to classify taxa from rare or occasional to nearly universal ones in control populations: Q25 (one or two samples), Q50 (three or four samples), Q75 (five or six samples), and Q100 (seven or eight samples). As for the samples from experimental populations, we focused only on the global composition at the family level, based on the aggregated samples at each time point. Due to the very small fraction of reads representing these nonbacterial communities, we did not use more complex computational tools for the annotation.

### Ecological and biostatistical analyses. (i) Alpha- and beta-diversity metrics estimation.

The alpha-diversity was based on Shannon and Chao1 indexes and estimated at the genus level using the microbiome R package (96). The Wilcoxon signed-rank test implemented in R software was used to evaluate differences in diversity metrics statistically. The beta-diversity analyses were performed by exploring the composition through canonical correspondence analyses (CCA), and the ADONIS test was used to identify statistically significant differences between groups. Both analyses are implemented in the vegan R package (97).

### (ii) Bacterial composition and taxon comparison between groups.

The bacterial composition comparisons between control and experimental conditions were performed at the genus level using ANCOM (analysis of composition of microbiomes) (98). This method allows for detecting differentially abundant taxa between groups. Barplots of the bacterial composition were plotted using the R package phyloseq (99).

### (iii) Self-organizing map (SOM) analysis.

Clustering analyses to classify selected bacteria according to their abundance profile were performed with the SOM package (100). The analyses applied a univariate scaling to the abundance matrix to obtain profiles with the same mean of 0 and a standard deviation of 1. The scaled profiles were clustered by creating a SOM with the different temporal dynamics of taxon groups. The clusters include taxa that behave similarly across time, meaning that they increase or decrease correspondingly. Based on the ANCOM results, bacteria with statistically significant differences compared to the control group (Ca+CCa versus Ka, KKn, and KKa) were selected for the longitudinal analyses.

### (iv) Core gut bacterial estimation.

The core bacteria were estimated at the genus level for control adults (C0a, C10a, C30a, CC0a, CC10a, and CC30a) and nymphs (CC22n and CC34n) separately. The microbiome R package (95) was employed for this purpose, using the “core_members” function with the following settings: detection, 0; prevalence, 0.99.

### (v) Cooccurrence network estimation of bacterial gut microbiome composition.

We used SparCC software (101) to detect cooccurrence networks based on bacterial composition. Networks were inferred for control conditions in adults (Ca+CCa) and nymphs (CCn). Correlation coefficients were estimated from genus abundance tables with 100 iterations. To consider a correlation as significant, we selected *P* values < 0.01 with 500 bootstraps. We used the igraph package implemented in R software to plot the networks and clusters (Newman-Girvan algorithm) and to display them (force-directed layout option) (102).

### Characterizing the functional potential and the resistome.

The functional annotation of metagenomes was based on KEGG pathway profiles. CCA and the ADONIS test implemented in the vegan R package (97) were applied to determine statistical differences between control and experimental groups. To identify pathways with relative differential abundance, we used ANCOM (98). Only KEGG pathways with a relative abundance of >0.25% per condition were included in the ANCOM analyses.

Functional characterization of antibiotic-resistant genes (ARGs) was carried out by aligning the reads against the functional antibiotic resistance element database (FARME DB) (103) with blastp (E value of 0.000001 and minimum percentage of identical matches of 90%). To perform a comprehensive analysis of the resistome of this insect species, we also included the metagenomes from a previous study of *B. germanica* under vancomycin and ampicillin treatments (13). The resistance gene reads were normalized to the total reads per sample (proportions) to estimate the abundance of resistance genes regarding the total gene content. The resistance gene reads were normalized to the total resistance genes reads per sample to compare the resistance gene profiles between samples.

### Data availability.

The raw sequence data generated in this study are available from the European Bioinformatics Institute (EBI), EMBL Nucleotide Archive under the study accession number PRJEB41306. Sequences are available under accession numbers ERS5335316 to ERS5335390.
