# Supplementary figures and images for: Interkingdom Gut Microbiome and Resistome of the Cockroach Blattella germanica
Source: mSystems. 2021 May 11;6(3):e01213-20. doi: 10.1128/mSystems.01213-20 (PMC8125077; doi:10.1128/mSystems.01213-20)

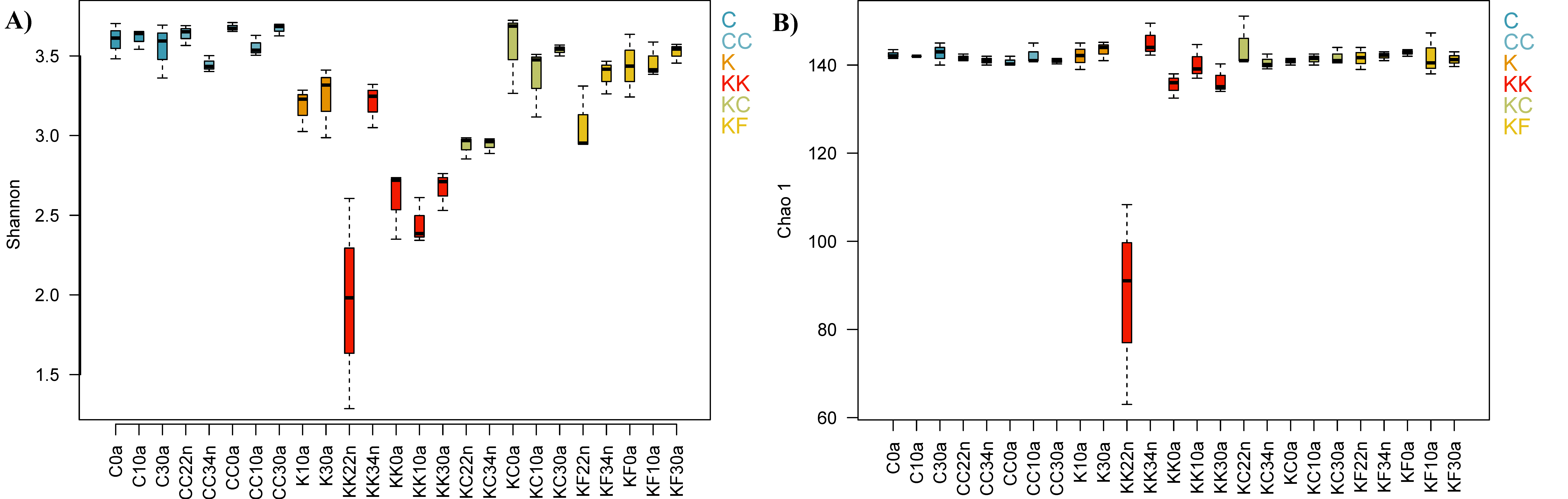

Supplement: FIG S2 [file mSystems.01213-20-sf002.tif]
